# Update Pathogenesis of COPD

**Published:** 2017

**Authors:** Ian Michael Adcock

**Affiliations:** National Heart & Lung Institute (NHLI), Imperial College London

The immunopathology of chronic obstructive pulmonary disease (COPD) is based on the innate and adaptive inflammatory immune responses to the chronic inhalation of cigarette smoking. In the last quarter of the century, the analysis of specimens obtained from the lower airways of COPD patients compared with those from a control group of age-matched smokers with normal lung function has provided novel insights on the potential pathogenetic role of the different cells of the innate and acquired immune responses and their pro/anti-inflammatory mediators and intracellular signaling pathways, contributing to a better knowledge of the immunopathology of COPD both during its stable phase and during its exacerbations. This also has provided a scientific rationale for new drugs discovery and targeting to the lower airways. This review summarises and discusses the immunopathology of COPD patients, of different severity, compared with control smokers with normal lung function.

